# Cuticular Hydrocarbons of Orchid Bees Males: Interspecific and Chemotaxonomy Variation

**DOI:** 10.1371/journal.pone.0145070

**Published:** 2015-12-29

**Authors:** Aline Borba dos Santos, Fábio Santos do Nascimento

**Affiliations:** Laboratório de Comportamento e Ecologia de Insetos Sociais. Departamento de Biologia, Faculdade de Filosofia, Ciências e Letras de Ribeirão Preto (FFCLRP), Universidade de São Paulo. Av. Bandeirantes, 3900. CEP 14040–901. Ribeirão Preto, São Paulo, Brazil; Salford University, UNITED KINGDOM

## Abstract

Recent studies have investigated the composition of compounds that cover the cuticle in social insects, but few studies have focused on solitary bees. Cuticular hydrocarbons may provide a tool for chemotaxonomy, and perhaps they can be used as a complement to morphology and genetic characters in phylogenetic studies. Orchid bees (Tribe Euglossini) are a highly diverse group of Neotropical bees with more than 200 species. Here, the cuticular hydrocarbons of 17 species were identified and statistical analysis revealed 108 compounds, which allowed for the taxonomic classification according to the genera. The most significant compounds discriminating the four genera were (Z)-9-pentacosene, (Z,Z)-pentatriacontene-3, (Z)-9-tricosene, and (Z)-9-heptacosene. The analyses demonstrated the potential use of CHCs to identify different species.

## Introduction

Orchid bees (Hymenoptera, Apidae, Euglossini) are endemic to the Neotropical region, and exhibit diverse behavioral syndromes that can be solitary, communal, primitively eusocial or cleptoparasitic [1]. These bees have a robust body with a bright tegument, long tongue, and modified tibiae in males to collect aromatic substances [2]. Such features together with other morphological features are used for identification [1]. The phylogenetic position of Euglossini within corbiculate bees has been debated because they are the only exception in the group that does not show an advanced eusociality [3].

As an auxiliary tool to classification, the cuticle chemical composition has been used as a taxonomic marker in several insects groups over the last 30 years [4,5]. The hydrocarbons covering the cuticle are synthesized from lipids by oenocytes [6] and these compounds, including *n*-alkanes and *n-*alkenes, frequently occur as a mix of isomers [7]. Among these compounds, alkadienes and alkatrienes are less common [8]. Hydrocarbons are promising compounds to be used in the chemical taxonomy identification of sister species that have not been previously separated [9]. A recent revision on CHCs in 241 hymenopteran species [10] stated that both solitary and social taxonomic groups produced almost all types of olefins and methylalkanes, which suggest that the majority of CHC classes and their associated biochemical pathways were already present early in Hymenoptera evolutionary history.

Beyond the taxonomic identification utility, the most known function of cuticular hydrocarbons (CHCs) is communication across species [11,12, 13]. CHCs act as sex pheromones, recognition cues among species, recognition between colonies, and sexual attractiveness signalers [14, 15, 16].

Bees make olfactory distinctions based on chemical cues. For instance, bees learn to discriminate between alkanes that differ by only two carbon atoms [17]. According to Breed & Stiller [18], alkanes are an interesting example of how the chemoreception system can operate. Studies honeybees have shown that alkenes along with other cuticular components, such as fatty acids, may be used to determine the division of tasks among individuals [19] or to recognize nestmates [20].

In ants, Martin *et al*. [21] compared the CHC data obtained in *Formica* with other data using genetic markers to mitochondrial DNA [22], allozymes [23], morphology, and behavior. They showed that CHCs have a genetic origin because these data were aligned with each other. Molecular and chemical analyses are complementary because the CHCs are the result of genetic inheritance. Thus, individuals of the same species have characteristics that are independent of the environment in which they live in [24].

In solitary bees, previous study reported the presence of sex pheromone on the cuticle chemical composition [21]. However, few studies have evaluated the composition of the cuticle lipids [8]. Some problems are frequently been found to obtain distinctness among male orchid bees. For example, the use of molecular markers showed difficulties, especially concerning related species, as have been found in cytochrome oxidase subunit 1 (COI) or microsatellite markers to separate the sibling species *Euglossa viridissima* Friese and *Eg*. *dilemma* Bembé & Eltz [25]. Chemical analyses using perfume compositions have provided relevant information for distinction among some species [26]. Nevertheless, as appointed by Pokorny *et al* [9] these compounds are gradually accumulated from the environment and may change between individuals.

In our study we analyzed the male chemical composition of cuticle in seventeen Euglossini species emphasizing the hydrocarbons and aiming to elucidate the potential of this tool to species identification into this tribe. Specifically, we aimed (1) to characterize the CHC profile of some Euglossini species, and from this (2) to quantify the differences among these species, (3) to identify how overall chemical distinctness relates to existing phylogeny, (4) to evaluate the potential of using CHC profiling as a diagnostic tool, and (5) to identify the compounds that allow this classification. We found that there is a chemical identity between all the species analyzed; these results support the hypothesis that CHCs can be used as chemotaxonomic tools.

## Materials and Methods

Males of orchid bees were collected in a protection area in Capela municipality in Sergipe state (10°46'S, 37°01'W), Brazil between January 2012 and December 2013. We attracted males by using chemical baits (cineole, eugenol and vanillin), which were separately soaked in cotton balls inside perforated plastic bottles [27]. Each bee was then captured when tried to land on the baits, put in individual vials, and sacrificed by cooling.

Later, seventeen species of Euglossini were identified: *Eufriesea atlantica* Nemésio, *Eufriesea surinamensis* Linnaeus, *Exaerete smaragdina* Guérin-Méneville, *Exaerete frontalis* Guérin-Méneville, *Eulaema niveofasciata* Friese, *Eulaema nigrita* Lepeletier, *Eulaema cingulata* Fabricius, *Eulaema atleticana* Nemésio, *Euglossa cordata* Linnaeus, *Euglossa fimbriata* Rebêlo & Moure, *Euglossa hemichlora* Cockerell, *Euglossa ignita* Smith, *Euglossa liopoda* Dressler, *Euglossa nanomelanotricha* Nemésio, *Euglossa pleosticta* Dressler, *Euglossa securigera* Dressler, and *Euglossa townsendi* Cockerell.

The compounds from males were extracted by individually adding 2 ml of 95% *n*-hexane (Mallinckrodt Chemicals) for 2 min, and the extracts were analyzed using a GCMS-QP2010 ultra SHIMADZU (DB-5MS, 30 m) with helium gas as the carrier (1 ml/min). The oven program included increasing the temperature from 150 to 300°C at a rate of 5°C/min and maintaining the final temperature for 15 min. To identify the double bonds in alkenes and alkadienes, the remaining extract was reacted with dimethyl disulfide (DMDS) following the methods of Carlson *et al*. [28]. The chromatograms were analyzed using *n*-alkane standards (Sigma Chemical Co.), and the quantification was based on the peak area. In addition to the standards, the NIST8.0 and FFNSC1.3 libraries as well as the Kovats index were used for analysis.

Peak areas were standardized to represent relative contributions, which were then square root transformed. From these data, a triangular similarity matrix based on the Bray-Curtis index was derived. A typical profile for each species was traced.

Species similarity (in relative proportion) was ordinated in two dimensions using nonmetric multidimensional scaling (MDS), in which deviations are expressed in terms of “stress”; values <0.15 indicate a good fit of the structure plotted. One-way similarity analysis (ANOSIM) was tested among the species [29]. This type of analysis compared differences among groups using the median values according to the Bray-Curtis dissimilarity measures multivariate data. In this analysis, the R values range from -1 to 1, with R = 0 indicating similarity and R = 1 indicating dissimilarity. Negative values are rare, and they indicate high dissimilarity within each group and among the groups [9]. The SIMPER analysis identified the compounds that differed among the species.

To evaluate the individual influence of hydrocarbons on species and the tendency of the species to separate by gender, a principal coordinate analysis (PCO) was used. Axes resulting from the PCOs were plotted to view the main compounds that separated the groups. PCO is a generalization of canonical analysis of principal coordinates, but it preserves different distances of similarity [30]. Centroid analysis calculates the distance between groups and the distance of centroids. Centroid analysis was used to verify the distance among the genera. PRIMER v.6.0 was used for all of the analyses.

The R software [31] was used for a cluster analysis to separate species according to the compounds in the cuticle, which permitted the identification of potential similarity and dissimilarity among the species. This analysis grouped the species in classes based on similarity degrees considering all variables.

## Results

The cuticle hydrocarbon analyses revealed 108 compounds in total, including saturated and unsaturated alkanes, alkenes, and alkadienes (S1 Table). These compounds ranged from 16 to 37 carbon atoms in chain length. The compounds that were common to all species were (Z)-9-nonacosene, tricosane, pentacosane, heptacosane, nonacosane, and hentriacontane (Table 1). The chemical diversity was higher in *Eulaema* and *Exaerete*.

**Table 1:** Relative contribution (%) of cuticular hydrocarbon composition in Euglossini males. Number of analyzed individuals in parenthesis.

Compounds	*Eufriesea atlantica* (01)	*Eufriesea surinamensis* (01)	*Exaerete smaragdina* (20)	*Exaerete frontalis* (14)	*Eulaema niveofasciata* (13)	*Eulaema nigrita* (20)	*Eulaema cingulata* (18)	*Eulaema atleticana* (13)	*Euglossa townsendi* (01)	*Euglossa securigera* (09)	*Euglossa pleosticta* (03)	*Euglossa nanomelanotricha* (01)	*Euglossa liopoda* (09)	*Euglossa ignita* (21)	*Euglossa hemichlora* (01)	*Euglossa fimbriata* (08)	*Euglossa cordata* (33)
Hexadecane	-	-	0.10 ± 0.22	-	-	-	-	0.01 ± 0.01	-	-	-	-	-	-	-	-	-
Heptadecane	-	-	0.02 ± 0.04	-		-	-	-	-	-	-	-	-	-	-	-	-
Octadecane	-	-	0.13 ± 0.29	0.07 ± 0.19	0.07 ± 0.21	0.00 ± 0.01	-	0.01 ± 0.01	-	0.50 ± 1.33	-	-	-	-	-	-	0.01 ± 0.05
Z-nonadecene	-	-	-	-	-	-	-	-	0.16	-	-	-	-	-	-	-	-
Nonadecane	0.07	-	-	0.01 ± 0.01	-	-	0.02 ± 0.02	0.02 ± 0.02	-	-	-	-	-	-	-	-	-
Eicosane	-	0.01	0.17 ± 0.36	0.08 ± 0.23	0.14 ± 0.32	0.02 ± 0.06	0.05 ± 0.14	0.09 ± 0.27	-	0.64 ± 1.65	-	-	-	-	-	-	0.03 ± 0.08
(Z)-11-heneicosene	-	-	-	-	-	-	-	0.78 ± 0.51	-	-	-	-	-	-	-	-	-
(Z)-9-henicosene	0.20	-	-	-	0.05 ± 0.07	-	-	0.07 ± 0.06	0.13	-	-	-	-	-	-	-	-
(Z)-7-henicosene	-	-	-	-	0.03 ± 0.04	-	-	-	-	-	-	-	-	-	-	-	-
Henicosane	0.05	-	0.09 ± 0.04	0.11 ± 0.07	0.69 ± 0.64	0.04 ± 0.04	0.02 ± 0.03	1.00 ± 0.58	1.30	-	-	-	0.06 ± 0.01	-	0.15	-	0.26 ± 1.07
(Z)-9-docosene	-	-	0.03 ± 0.08	-	0.13 ± 0.13	-	-	0.14 ± 0.10	-	-	-	-	-	-	-	-	-
Docosane	0.55	0.02	0.15 ± 0.34	0.10 ± 0.25	0.21 ± 0.34	0.06 ± 0.03	0.06 ± 0.21	0.18 ± 0.30	0.28	0.77 ± 1.72	0.39 ± 0.35	-	0.11 ± 0.08	-	0.07	-	0.08 ± 0.15
(Z)-9-tricosene	-	-	0.20 ± 0.17	0.06 ± 0.05	26.33 ± 15.81	1.02 ± 0.51	0.02 ± 0.02	18.99 ± 12.71	-	-	1.65 ± 0.61	-	1.76 ± 1.59	-	-	-	0.03 ± 0.06
(Z)-7-tricosene	-	-	0.30 ± 0.19	0.03 ± 0.04	-	1.06 ± 2.41	0.01 ± 0.03	0.42 ± 0.31	-	-	-	-	-	-	-	-	-
(Z)-5-tricosene	-	-	-	-	0.93 ± 2.92	-	-	0.01 ± 0.01	13.81	-	-	-	-	-	-	-	-
Tricosane	0.67	0.30	2.00 ± 1.04	0.18 ± 0.08	4.27 ± 1.84	8.46 ±2.13	0.18 ± 0.15	6.13 ± 2.81	21.74	0.84 ± 0.36	0.75 ± 0.16	4.77	1.82 ± 0.82	1.13 ± 0.86	7.66	1.64 ± 2.63	0.13 ± 0.46
9-MeC_23_	-	-	0.08 ± 0.31	-	-	-	-	-	-	-	-	-	-	-	-	-	-
7-MeC_23_	-	-	0.02 ± 0.01	-	-	-	-	-	-	-	-	-	-	-	-	-	-
5-MeC_23_	-	-	0.01 ± 0.02	-	-	-	-	-	-	-	-	-	-	-	-	-	-
3-MeC_23_	-	-	0.01 ± 0.01	-	-	-	-	-	-	-	-	-	4.58 ± 4.48	-	-	-	4.29 ± 1.99
(Z)-11-tetracosene	-	-	-	-	0.10 ± 0.12	-	-	-	-	-	-	-	-	-	-	-	-
(Z)-9-tetracosene	-	-	-	-	0.12 ± 0.37	2.76 ± 0.96	-	0.15 ± 0.10	-	-	-	-	-	-	-	-	-
(Z)-7-tetracosene	-	-	0.02 ± 0.06	-	-	-	-	-	-	-	-	-	-	-	-	-	-
Tetracosane	0.19	0.10	0.18 ± 0.35	0.09 ± 0.26	0.29 ± 0.27	2.37 ± 7.53	0.10 ± 0.07	0.36 ± 0.27	0.34	0.87 ± 1.81	0.23 ± 0.08	-	30.80 ± 30.09	1.11 ± 1.86	0.48	0.77 ± 1.34	0.43 ± 0.17
4-MeC_24_	-	-	-	-	-	-	-	-	-	0.13 ± 0.20	-	-	-	-	-	-	-
5-MeC_24_	-	-	0.04 ± 0.06	-	-	-	-	0.04 ± 0.06	-	-	-	-	-	-	-	-	-
3-MeC_23_	-	-	-	-	0.01 ± 0.02	-	-	-	-	-	-	-	-	-	0.64	-	0.07 ± 0.15
(Z)-9-pentacosene	0.67	2.06	0.03 ± 0.04	0.08 ± 0.06	1.19 ± 0.61	52.75 ± 12.92	0.05 ± 0.04	2.16 ± 1.04	0.76	0.21 ± 0.22	-	0.70	4.21 ± 3.65	0.39 ± 0.41	0.34	0.17 ± 0.12	0.22 ± 0.12
(Z)-7-pentacosene	0.23	0.32	0.44 ± 0.32	-	0.28 ± 0.32	1.30 ± 3.46	1.71 ± 4.79	0.10 ± 0.04	-	0.25 ± 0.16	0.99 ± 0.43	-	0.41 ± 0.13	0.04 ± 0.05	-	-	0.00 ± 0.02
Pentacosane	12.58	9.23	2.34 ± 1.26	0.19 ± 0.08	9.05 ± 3.29	13.11 ± 3.52	8.08 ± 4.37	8.42 ± 1.55	13.03	11.91 ± 2.16	15.14 ± 1.68	17.56	6.91 ± 6.87	9.19 ± 2.19	13.01	13.97 ± 1.49	14.65 ± 1.80
(Z)-3-hexacosene	-	-	-	-	-	-	-	-	-	-	-	-	-	-	-	-	0.02 ± 0.08
11-MeC_25_; 13-MeC_25_	-	-	0.04 ± 0.02	0.02 ± 0.02	-	-	-	-	-	-	-	-	-	-	-	-	-
(Z)-9-hexacosene	0.52	1.28	0.05 ± 0.04	-	1.05 ± 2.90	-	1.35 ± 3.77	0.23 ± 0.12	0.15	0.05 ± 0.07	-	-	0.80 ± 0.67	0.38 ± 0.25	0.53	-	0.12 ± 0.17
(Z)-7-hexacosene	-	-	-	-	-	-	-	-	-	-	-	-	0.33 ± 0.14	-	-	-	-
(Z)-5-hexacosene	-	-	0.01 ± 0.01	-	-	-	-	-	-	-	-	-	-	-	-	-	-
Hexacosane	0.45	0.18	0.34 ± 0.34	0.12 ± 0.27	0.31 ± 0.33	0.28 ± 0.10	6.68 ± 15.45	0.23 ± 0.24	0.27	0.19 ± 0.09	0.21 ± 0.06	0.70	0.17 ± 0.16	0.95 ± 1.70	-	0.31 ± 0.37	0.14 ± 0.10
4-MeC_26_	-	-	-	-	-	-	-	-	-	0.16 ± 0.22	-	-	-	-	-	-	-
3-MeC_25_	-	-	-	-	-	-	-	-	0.72	-	-	-	-	-	-	-	-
(Z,Z)-heptacosadiene—1	-	-	0.01 ± 0.03	-	1.45 ± 2.24	-	-	0.31 ± 0.25	-	-	-	-	-	-	-	-	-
(Z)-3-heptacosene	-	-	-	-	0.40 ± 1.30	-	-	-	-	-	-	-	-	-	-	-	-
(Z)-11-heptacosene	-	51.36	-	-	-	-	-	5.63 ± 2.00	-	-	-	-	-	-	-	-	-
(Z)-9-heptacosene	22.56	1.93	4.56 ± 3.94	-	7.55 ± 6.65	1.40 ± 1.09	15.38 ± 15.0	0.30 ± 0.15	9.36	13.65 ± 3.81	3.08 ± 0.75	-	21.42 ± 19.65	18.85 ± 8.80	26.76	10.16 ± 6.31	20.56 ± 5.09
(Z)-7-heptacosene	1.79	0.13	0.41 ± 0.22	0.07 ± 0.09	2.89 ± 6.16	0.29 ± 0.94	5.34 ± 7.42	0.04 ± 0.04	0.15	10.97 ± 7.20	1.88 ± 0.52	22.90	13.40 ± 11.79	2.40 ± 1.43	0.53	9.36 ± 11.26	-
(Z)-5-heptacosene	-	-	-	-	0.06 ± 0.06	-	-	-	-	-	-	-	-	-	-	-	0.80 ± 0.87
Heptacosane	15.28	4.64	7.79 ± 3.99	0.31 ± 0.40	4.14 ± 1.06	4.78 ± 1.40	5.67 ± 3.20	2.79 ± 0.79	9.43	3.45 ± 0.91	6.48 ± 0.17	3.88	2.45 ± 1.08	3.92 ± 1.67	1.89	3.99 ± 0.86	2.94 ± 0.62
(Z,Z)-octacosadiene—1	-	-	-	0.04 ± 0.03	0.08 ± 0.10	-	-	0.12 ± 0.05	-	-	-	0.65	-	-	-	-	1.34 ± 0.34
(Z)-9-octacosene	1.21	0.60	0.15 ± 0.10	-	0.87 ± 0.36	-	1.66 ± 1.18	0.65 ± 0.22	0.64	1.07 ± 0.28	0.75 ± 0.21	-	0.25 ± 0.12	2.15 ± 1.22	1.69	0.99 ± 0.34	-
Octacosane	0.27	0.08	0.30 ± 0.31	0.09 ± 0.20	1.44 ± 4.10	0.22 ± 0.08	3.70 ± 13.98	0.16 ± 0.21	0.13	-	-	-	-	-	-	-	-
(Z,Z)-nonacosadiene—1	-	-	0.08 ± 0.12	0.27 ± 0.21	2.55 ± 3.19	-	-	4.59 ± 2.06	-	-	-	-	-	-	-	-	-
(Z,Z)-nonacosadiene—2	-	-	-	-	0.23 ± 0.26	-	-	1.43 ± 0.75	-	-	-	-	-	-	-	-	-
(Z,Z)-nonacosadiene—3	-	-	-	-	-	-	-	0.16 ± 0.12	-	-	-	-	-	-	-	-	-
Nonacosene–1	0.08	0.07	-	-	-	-	-	-	-	-	-	-	0.12 ± 0.05	-	-	-	-
(Z)-9-nonacosene	31.03	15.98	6.83 ± 4.39	0.28 ± 0.30	18.23 ± 10.81	0.22 ± 0.25	30.47 ± 16.84	15.08 ± 4.71	22.73	41.09 ± 9.69	48.60 ± 2.11	37.77	5.57 ± 3.29	51.46 ± 14.48	42.20	40.40 ± 13.60	46.01 ± 7.17
(Z)-7-nonacosene	0.88	0.68	0.36 ± 0.44	0.07 ± 0.08	0.80 ± 1.25	0.24 ± 0.90	-	-	0.30	3.36 ± 1.56	0.66 ± 0.11	-	0.47 ± 0.47	-	-	5.35 ± 2.00	-
(Z,Z)-5,9-nonacosene	-	-	-	-	-	-	-	-	-	-	-	-	-	-	-	-	0.63 ± 1.01
Nonacosane	6.15	2.14	2.81 ± 1.50	0.85 ± 0.92	2.45 ± 1.00	3.98 ± 1.89	3.58 ± 1.87	2.80 ± 0.71	2.31	3.89 ± 0.97	2.65 ± 0.41	5.31	1.91 ± 0.85	2.63 ± 1.18	1.89	4.18 ± 1.30	3.50 ± 0.77
(Z,Z)-tricontadiene– 1	-	-	-	-	-	0.06 ± 0.07	-	0.26 ± 0.17	-	-	-	-	-	-	-	-	-
(Z,Z)-tricontadiene– 2	-	-	-	-	-	0.37 ± 1.20	-	0.14 ± 0.17	-	-	-	-	-	-	-	-	-
(Z)-9-triacontene	0.31	0.18	0.19 ± 0.19	-	0.49 ± 0.29	0.02 ± 0.05	0.85 ± 0.58	0.39 ± 0.26	-	0.61 ± 0.59	-	-	-	0.48 ± 0.25	-	0.44 ± 0.30	0.21 ± 0.17
(Z)-8-triacontene	-	-	0.03 ± 0.02	-	-	-	-	-	-	-	-	-	-	-	-	-	-
Triacontane	0.08	0.09	0.19 ± 0.28	0.11 ± 0.18	0.36 ± 0.58	0.19 ± 0.08	0.95 ± 2.99	0.16 ± 0.18	-	0.61 ± 1.34	0.04 ± 0.06	-	0.05 ± 0.01	0.72 ± 1.35	-	0.49 ± 0.95	0.05 ± 0.08
Me-C_27_	0.27	0.26	-	-	-	-	-	-	-	0.55 ± 0.48	-	-	0.28 ± 0.17	-	0.14	-	-
(Z,Z)-hentriacontadiene—1	-	-	0.21 ± 0.21	-	0.94 ± 1.07	-	-	5.15 ± 2.09	-	-	0.30 ± 0.21	-	-	-	-	-	-
(Z,Z)-hentriacontadiene—2	-	-	0.22 ± 0.21	-	0.33 ± 0.92	-	-	1.87 ± 1.92	-	-	-	-	-	-	-	-	-
(Z.Z)-hentriacontadiene—3	-	-	-	-	-	-	1.60 ± 4.93	0.89 ± 1.59	-	-	-	-	-	-	-	-	-
(Z)-15-hentriacontene	-	-	-	-	0.46 ± 0.89	-	-	-	-	-	-	-	-	-	-	-	-
(Z)-11-hentriacontene	-	-	0.35 ± 0.79	0.20 ± 0.42	-	-	-	-	-	-	-	-	-	-	-	-	-
(Z)-10-hentriacontene	-	-	-	-	-	-	-	-	-	1.95 ± 0.57	-	-	-	-	-	-	-
(Z)-9-hentriacontene	1.79	5.23	6.29 ± 5.71	1.74 ± 1.64	3.78 ± 1.88	0.30 ± 0.80	6.07 ± 4.00	1.00 ± 1.47	-	0.16 ± 0.13	7.82 ± 5.53	2.17	1.42 ± 1.09	0.05 ± 0.07	-	1.91 ± 1.05	2.41 ± 0.67
(Z)-7-hentriacontene	0.29	0.17	1.80 ± 0.73	1.62 ± 0.68	0.60 ± 1.41	-	0.41 ± 1.54	6.49 ± 4.77	1.68	-	-	1.01	-	3.11 ± 1.58	1.62	2.73 ± 1.24	0.08 ± 0.23
Hentriacontane	1.60	2.27	1.51 ± 0.91	2.63 ± 0.66	2.72 ± 1.71	3.24 ± 1.01	3.20 ± 1.59	2.01 ± 0.76	0.55	1.18 ± 0.55	7.73 ± 9.85	2.16	0.52 ± 0.28	1.04 ± 0.57	0.41	1.25 ± 0.63	0.92 ± 0.39
11-MeC_23_ (158/9; 308/9); 13-MeC_31_ (196/7; 281/2); 15-MeC_31_ (224/5; 252/3)	-	-	-	-	-	-	-	0.20 ± 0.24	-	-	-	-	-	-	-	-	-
(Z,Z)-dotriacontadiene—1	-	-	0.12 ± 0.10	0.08 ± 0.05	-	-	0.63 ± 0.57	0.57 ± 0.53	-	-	-	-	-	-	-	-	-
(Z,Z)-dotriacontadiene—2	-	-	0.10 ± 0.05	-	-	-	-	0.23 ± 0.32	-	-	-	-	-	-	-	-	-
(Z)-11-dotriacontene	-	-	0.34 ± 0.19	0.45 ± 0.09	0.12 ± 0.16	-	0.26 ± 0.22	0.08 ± 0.08	-	-	-	-	-	-	-	-	-
(Z)-9-dotriacontene	-	-	0.07 ± 0.05	0.16 ± 0.04	-	-	0.00 ± 0.01	-	-	-	-	-	-	-	-	-	-
Dotriacontane	-	0.04	0.15 ± 0.26	0.16 ± 0.16	0.14 ± 0.23	0.08 ± 0.08	0.05 ± 0.03	0.09 ± 0.16	-	0.44 ± 1.11	-	-	-	-	-	-	-
(Z,Z)-tritriacontadiene—1	-	-	0.69 ± 0.58	0.76 ± 0.91	0.30 ± 0.35	-	0.06 ± 0.21	2.51 ± 1.67	-	-	-	-	-	-	-	-	-
(Z,Z)-tritriacontadiene—2	-	-	6.41 ± 2.92	3.24 ± 3.02	-	-	0.14 ± 0.53	0.72 ± 1.41	-	-	-	-	-	-	-	-	0.01 ± 0.04
(Z,Z)-tritriacontadiene—3	-	-	5.65 ± 2.07	4.39 ± 1.49	-	-	-	-	-	-	-	-	-	-	-	-	0.05 ± 0.14
(Z,Z)-tritriacontadiene—4	-	-	0.60 ± 0.48	1.36 ± 0.32	-	-	-	-	-	-	-	-	-	-	-	-	-
(Z)-14-tritriacontene	-	-	-	-	0.22 ± 0.20	-	-	-	-	-	-	-	-	-	-	-	-
(Z)-11-tritriacontene	-	-	-	-	-	-	-	0.86 ± 0.69	-	-	-	-	-	-	-	-	-
(Z)-9-tritriacontene	0.15	0.37	11.60 ± 3.10	17.32 ± 2.29	0.34 ± 0.23	-	0.63 ± 0.60	0.64 ± 0.81	-	-	0.31 ± 0.17	-	0.15 ± 0.09	-	-	-	-
(Z)-7-tritriacontene	-	-	3.41 ± 1.95	5.19 ± 1.05	-	-	0.03 ± 0.12	0.70 ± 0.75	-	-	-	-	-	-	-	-	-
(Z)-5-tritriacontene	-	-	-	-	-	-	-	0.05 ± 0.10	-	-	-	-	-	-	-	-	-
Tritriacontane	0.11	0.26	1.15 ± 0.99	4.12 ± 1.43	0.45 ± 0.57	0.58 ± 0.62	0.28 ± 0.15	0.45 ± 0.20	-	0.15 ± 0.08	0.36 ± 0.41	0.41	0.03 ± 0.03	-	-	-	-
(Z,Z)-tetratriacontadiene—1	-	-	0.07 ± 0.12	0.12 ± 0.09	-	-	0.33 ± 0.32	-	-	-	-	-	-	-	-	-	-
(Z,Z)-tetratriacontadiene—2	-	-	0.13 ± 0.10	0.39 ± 0.09	0.13 ± 0.19	0.78 ± 0.54	-	-	-	-	-	-	-	-	-	-	-
(Z,Z)-tetratriacontadiene—3	-	-	0.45 ± 0.25	0.98 ± 0.26	-	-	-	-	-	-	-	-	-	-	-	-	-
(Z,Z)-tetratriacontadiene—4	-	-	0.13 ± 0.08	0.26 ± 0.11	-	-	-	-	-	-	-	-	-	-	-	-	-
Tetratriacontane	-	-	0.11 ± 0.18	0.09 ± 0.12	0.11 ± 0.21	-	-	-	-	0.33 ± 0.78	-	-	-	-	-	-	-
(Z,Z)-pentatriacontadiene—1	-	-	0.10 ± 0.28	-	-	-	0.01 ± 0.02	0.29 ± 0.27	-	-	-	-	-	-	-	-	-
(Z,Z)-pentatriacontadiene—2	-	-	0.77 ± 0.73	0.14 ± 0.33	-	-	0.03 ± 0.07		-	-	-	-	-	-	-	-	-
(Z,Z)-pentatriacontadiene—3	-	-	18.05 ± 9.28	30.76 ± 6.61	-	-	0.02 ± 0.04	-	-	-	-	-	-	-	-	-	-
(Z,Z)-pentatriacontadiene—4	-	-	4.62 ± 3.01	8.29 ± 1.56	-	-	0.03 ± 0.07	-	-	-	-	-	-	-	-	-	-
(Z)-pentatriacontene– 1	-	-	-	-	-	-	-	0.64 ± 0.65	-	-	-	-	-	-	-	1.88 ± 2.48	-
(Z)-10-pentatriacontene	-	-	3.15 ± 2.46	-	-	-	-	-	-	-	-	-	-	-	-	-	-
(Z)-9-pentatriacontene	-	-	0.27 ± 0.26	8.81 ± 2.90	-	-	0.01 ± 0.03	-	-	-	-	-	-	-	-	-	-
(Z)-7-pentatriacontene	-	-	-	-	-	-	0.25 ± 0.88	-	-	-	-	-	-	-	-	-	-
Pentatriacontane	-	-	0.08 ± 0.18	0.66 ± 0.48	0.01 ± 0.02	-	-	0.03 ± 0.02	-	-	-	-	-	-	-	-	-
(Z,Z)-hexatriacontadiene—1	-	-	-	0.15 ± 0.12	-	-	-	-	-	-	-	-	-	-	-	-	-
Hexatriacontane	-	-	0.06 ± 0.11	-	0.11 ± 0.24	-	-	0.01 ± 0.01	-	-	-	-	-	-	-	-	-
(Z,Z)-heptacontatriacontadiene—1	-	-	-	1.90 ± 0.90	-	-	-	-	-	-	-	-	-	-	-	-	-
(Z,Z)-heptacontatriacontadiene—2	-	-	-	0.78 ± 0.53	-	-	-	-	-	-	-	-	-	-	-	-	-
(Z)-10-heptatriacontene	-	-	0.22 ± 0.20	-	-	-	-	-	-	-	-	-	-	-	-	-	-
(Z)-9-heptatriacontene	-	-	0.28 ± 0.49	-	-	-	-	-	-	-	-	-	-	-	-	-	-
(Z)-7-heptatriacontene	-	-	0.31 ± 0.34	-	-	-	-	-	-	-	-	-	-	-	-	-	-

(-)–unidentified compound; (Z)–insaturation; (Me)–Methyl group

**Fig 1:**
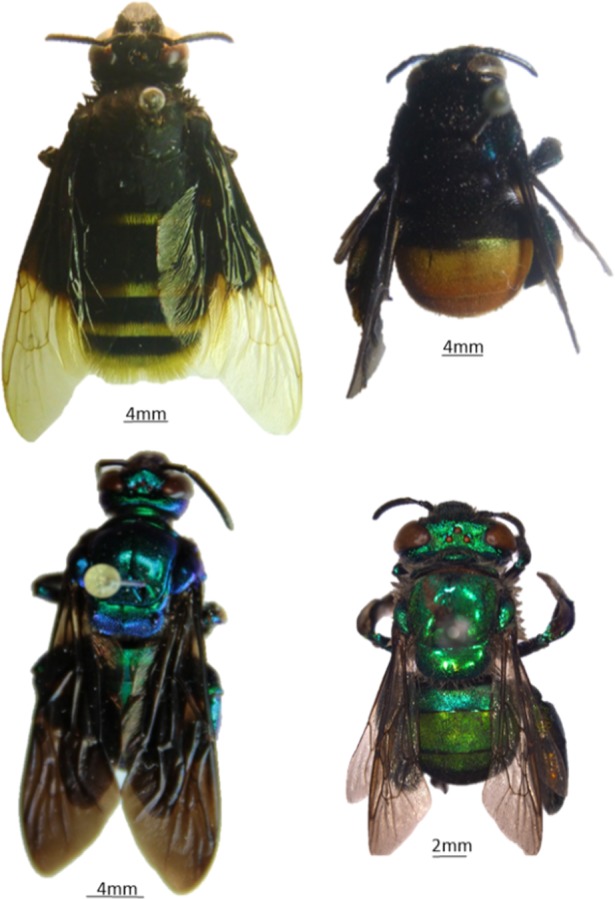
Euglossini bee Genera. A–*Eulaema* (*Eulaema atleticana*); B–*Eufriesea* (*Eufriesea surimanensis*); C–*Exaerete* (*Exaerete frontalis*); D–*Euglossa* (*Euglossa pleosticta*).

Among the four genera analyzed (Fig 1), *Exaerete* was the only that showed the following exclusive compounds in all of the species identified: one (Z,Z)-C_33_ compound and two (Z,Z)-C_34_ compounds. The other genera exhibited compounds that varied (presence/absence) among the species. *Exaerete* had a higher proportion of alkadienes (*Ex*. *frontalis*: 33.33% and *Ex*. *smaragdina*: 25%).

*Eulaema* had the second highest composition of alkadienes, and alkadienes were absent in *Eufriesea*. Moreover, some *Euglossa* species had the highest percentages of methyl groups. Alkane and alkene were common to all species, with an average percentage composition of 42 and 44%, respectively.

The variability of compounds in different species, evaluated by the permutation test through ANOSIM, revealed significant differences among genera (R = 0.635 and P<0.02). To examine the hypothesis of significant differences in the genera, the similarity analysis showed that the major differences were between *Eufriesea* x *Exaerete* (R = 1.0 and P<33.1) and *Euglossa* x *Exaerete* (R = 1.0 and P<1.8).

The compounds that mostly contributed to the similarity and dissimilarity within and between the groups formed in the cluster analysis were defined by the similarity percentages (SIMPER). With regard to the diagnoses within each group, the major similarity occurred in *Eufriesea* species (67.12%), followed by *Exaerete* (65.50%), *Euglossa* (65.14%) and *Eulaema* (52.51%).

The dissimilarity percentage between the groups was higher between *Exaerete* and *Euglossa* (70.69%), which was primarily due to the presence of the alkene double bond in the 9 position. The remaining dissimilarity percentages between groups in descending order were *Eufriesea* and *Exaerete* (66.34%), *Exaerete* and *Eulaema* (62.89%), *Eulaema* and *Euglossa* (48.43%), *Eufriesea* and *Eulaema* (44.30%), and *Eufriesea* and *Euglossa* (36.86%).

**Fig 2:**
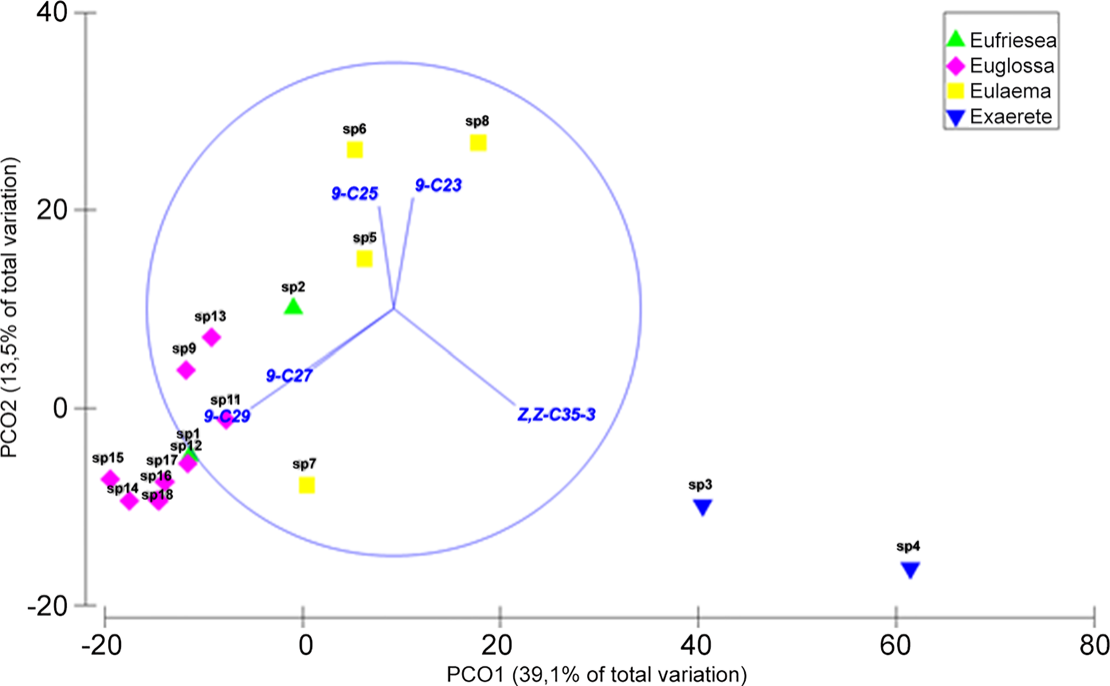
PCO pf cuticular hydrocarbon compounds of all Euglossini male genera studied. (sp1)—*Ef*. *atlantica*; (sp2)—*Ef*. *surinamensis*; (sp3)—*Ex*. *smaragdina*; (sp4)—*Ex*. *frontalis*; (sp5)—*El*. *niveofasciata*; (sp6)—*El*. *nigrita*; (sp7)—*El*. *cingulata*; (sp8)—*El*. *atleticana*; (sp9)—*Eg*. *townsendi*; (sp10)—*Eg*. *securigera*; (sp11)—*Eg*. *pleosticta*; (sp12)—*Eg*. *nanomelanotricha*; (sp13)—*Eg*. *liopoda*; (sp14)—*Eg*. *ignita*; (sp15)—*Eg*. *hemichlora*; (sp16)—*Eg*. *fimbriata*; (sp17)—*Eg*. *cordata*.

The MDS “stress” was 0.1, which showed that the ordination recovered the main patterns of cuticle composition and suggested that there was a tendency of proximity between individuals of the same genus, considering the Bray-Curtis similarity. The most distinct species was *Ex*. *smaragdina*. Similar results were obtained with the PCO analysis (Fig 2), which showed that the individuals formed groups related to gender, except for *Eufriesea*. This analysis suggested that 52.6% of the variation could be explained by the two first axes and that the most important compounds for separation were as follows: (Z)-9-pentacosene, (Z,Z)-pentatriacontene-3, (Z)-9-tricosene, and (Z)-9-heptacosene.

**Fig 3:**
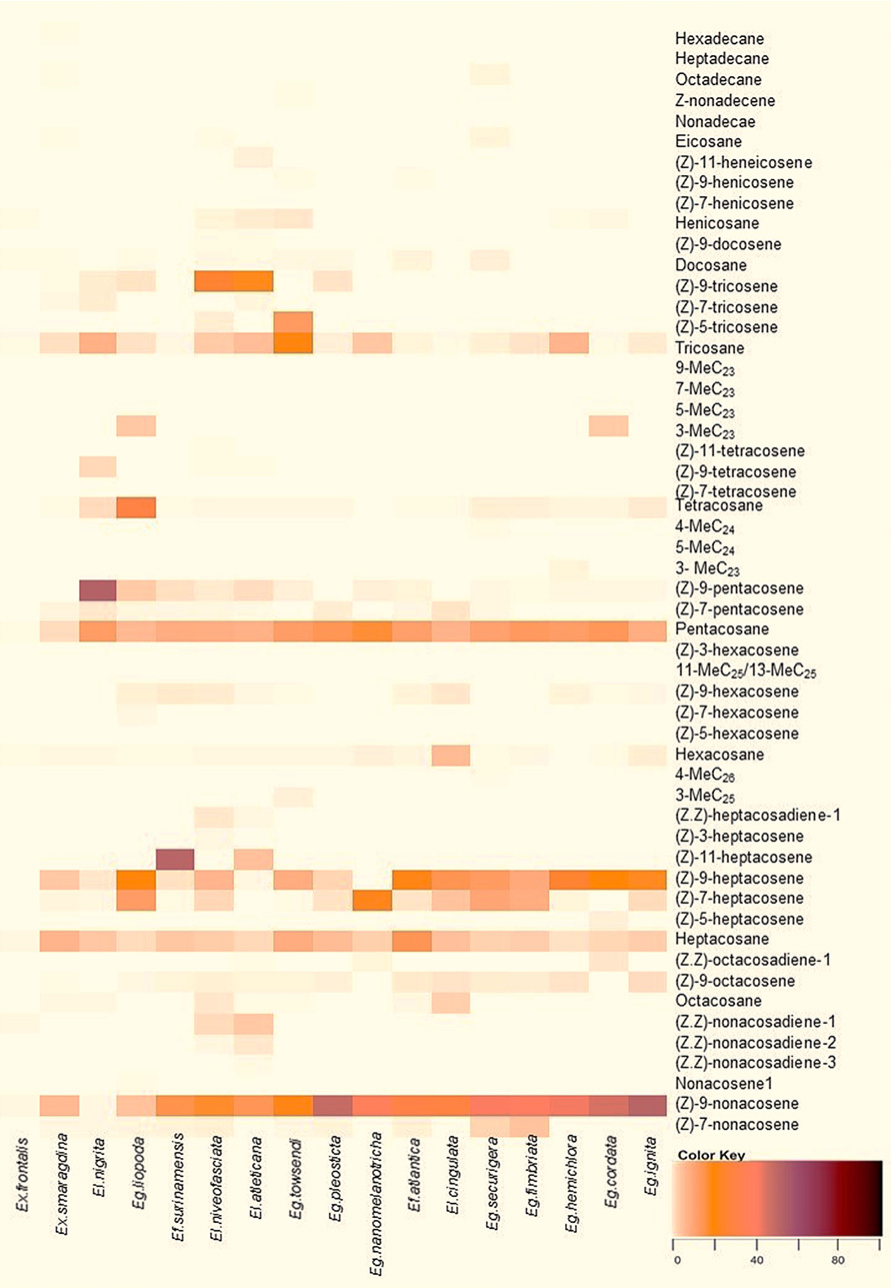
CHCs in Euglossini males according to the similarity and dissimilarity among them. The color scale represents the relative percentage of a compound contributed to the total area of each peak.

The previously performed analysis was represented by cluster analysis using all of the identified hydrocarbons. The heatmap analysis showed the presence or absence of compounds in addition to the peak area percentage in the chromatograms for each species (Fig 3).

The centroids represented the chemical distance among the genera. A higher distinction in *Exaerete* and *Eulaema* was corroborated by the qualitative analysis, and the same results were found in the MDS and cluster analyses in which *El*. *cingulata* was chemically more similar to *Euglossa* species (Table 1).

## Discussion

Our data allowed the accurate characterization of each species according to the cuticle chemical profile. For instance, the class of CHCs present in greater proportions among the compounds was alkenes with a double bond at 9 and 7 positions, respectively. Males of Euglossini may also use alkenes as short-range sex pheromones during courtship contests such as other bees [10]. On the contrary, the low amount of methyl-branched alkanes of these males can be related with the absence of a nestmate recognition system in male bees. *Exaerete* males presented exclusive alkadienes which can be considered a synapomorphy such as its cleptoparasite behavior [2].

The level of n-alkanes, which were the second most abundant compounds in this study, has been associated with arid environments that have high temperatures [32]. *N*-alkanes have been considered the best hydrocarbons for waterproofing properties [33]. Thus, these compounds may be present more for the control of water loss than for communication between individuals.

However, methyl-branched alkanes have been indicated as chemotaxonomic markers in Diptera [34], Hemiptera [35], Isoptera [36], Orthoptera [37], and several Hymenoptera reviewed in [38]. In the later example, by analyzing the cuticular profile of two sibling *Euglossa* species, Pokorny *et al*. [9] verified that the CHCs are potentially useful tools for the chemical taxonomy because they had clear variations on their cuticular chemical profiles that allowed distinguishing two cryptic species accordingly. Indeed, equally important to species recognition is genus differentiation [39]. Here, we used seventeen species distributed into four genera of Euglossini, and all analyses indicated a differentiation among them through the use of CHCs with a clearer distinction when genera were identified. The cuticular hydrocarbon diversity across species partly reflects the variation of the environment in which they live, food resources and their life style.

There are several hypotheses regarding the relationship among Euglossini species, their classification within five genera, and advanced analysis based on molecular and morphological characters [40]. Concerning the hydrocarbon composition, species that belong to the same genus are qualitatively similar to each other [9], which can be verified in this study and in other Hymenoptera [10].

In conclusion, the analyses of cuticular hydrocarbon profiles in seventeen species of Euglossini showed that the accurate identification of different species through the use of cuticular compounds is possible. Despite the presence of compounds common to all individuals, each species presented a characteristic chemical profile that can be used as taxonomic characters.

## Supporting Information

S1 Table Data set of males analyzed in this study. (XLSX)
